# Optimization of simultaneous microwave/ultrasonic-assisted extraction of phenolic compounds from walnut flour using response surface methodology

**DOI:** 10.1080/13880209.2017.1347189

**Published:** 2017-07-25

**Authors:** Yan Luo, Wanxing Wu, Dan Chen, Yuping Lin, Yage Ma, Chaoyin Chen, Shenglan Zhao

**Affiliations:** a Faculty of Traditional Chinese Medicine, Yunnan University of Traditional Chinese Medicine, Kunming, China;; b Faculty of Life Science, Kunming University of Science and Technology, Kunming, China;; c The Chemical Analysis Division, Yunnan Institute of Tobacco Quality Inspection and Supervision, Kunming, China

**Keywords:** Walnut polyphenol, central composite design (CCD), total polyphenol yield (TPY), constituent identification, LC–MS

## Abstract

**Context:** Walnut is a traditional food as well as a traditional medicine recorded in the Chinese Pharmacopoeia; however, the large amounts of walnut flour (WF) generated in walnut oil production have not been well utilized.

**Objective:** This study maximized the total polyphenolic yield (TPY) from the walnut flour (WF) by optimizing simultaneous ultrasound/microwave-assisted hydroalcoholic extraction (SUMAE).

**Materials and methods:** Response surface methodology was used to optimize the processing parameters for the TPY, including microwave power (20–140 W), ultrasonic power (75–525 W), extraction temperature (25–55 °C), and time (0.5–9.5 min). The polyphenol components were analysed by LC–MS.

**Results:** A second-order polynomial model satisfactorily fit the experimental TPY data (*R*^2^ = 0.9932, *P* < 0.0001 and *R*_adj_^2^    = 0.9868). The optimized quick extraction conditions were microwave power 294.38 W, ultrasonic power 93.5 W, temperature 43.38 °C and time 4.33 min, with a maximum TPY of 34.91 mg GAE/g, which was a rapid extraction. The major phenolic components in the WF extracts were glansreginin A, ellagic acid, and gallic acid with peak areas of 22.15%, 14.99% and 10.96%, respectively, which might be used as functional components for health food, cosmetics and medicines.

**Discussion and conclusion:** The results indicated that walnut flour, a waste product from the oil industry, was a rich source of polyphenolic compounds and thus could be used as a high-value functional food ingredient.

## Introduction

Walnut is one of the most popular tree nuts worldwide because of its nutritional, health and sensory attributes (Martinez et al. 2010). The global production of walnut was approximately 3.5 million tons in 2013, China had become the leading world producer (1.7 MT), followed by Iran (0.45 MT) and USA (0.42 MT) (FAO 2013). Walnut kernel is a normal food as well as a traditional medicine recorded in the Chinese Pharmacopoeia (China 2015). Previous studies indicated that walnut is rich in oil (50–70%), proteins (14–24%), tocopherol and phenolic compounds (Gomez-Caravaca et al. 2008; Miraliakbari and Shahidi 2008). Most phenolic compounds commonly identified in walnut are phenolic acids, condensed tannins, ellagic acid and flavonoids, which potentially have free radical-scavenging capacities and have a protective effect on the susceptibility of LDL-cholesterol to oxidative modification *in vitro* (Kris-Etherton et al. 1999; Anderson et al. 2001; Li et al. 2006).

Owing to its high contents of oil and essential fatty acid, the walnut is a traditional source of edible oil. The walnut flour (WF) remaining from walnut oil production has commonly used as manure or animal feed (Liu and Jiang 2014; Wu et al. 2014). Considering the WF to be rich source of phenolic compounds (Martinez et al. 2010), effective exploitation of WF could not only promote the economic value of walnut but also protect the environment. However, few studies to date have focused on the recovery of phenolic compounds in WF.

Extraction is thought to be an important stage in the use of phenolic compounds (Lapornik et al. 2005). Conventional extraction methods of phenolic compounds have some limitations including high-solvent consumption, long required extraction times and the possible degradation of target compounds. To overcome these drawbacks, several techniques have been developed for the extraction of natural antioxidants from plants, including ultrasound-assisted extraction (Yue et al. 2012; Kazemi et al. 2016), pulsed ohmic heating extraction (Darra et al. 2012), microwave-assisted extraction (Mishra and Aeri 2016) and simultaneous microwave/ultrasound-assisted extraction (Ghafoor et al. 2009; Pingret et al. 2012). The combination of ultrasound and microwave offers an intriguing method to focalize two different sources of energy, with their own specific effects. Currently simultaneous ultrasound/microwave-assisted extraction (SUMAE) has seen wide use (Zhang and Liu 2008; Nayak et al. 2015), this technique exhibits very high-efficiency due to the synergetic effect induced by the heat transfer enhancement of microwave and the mass transfer enhancement of ultrasound. Thus, the application of SUMAE in the extraction of polyphenols may greatly shorten preparation time and enhance production efficiency.

Response surface methodology (RSM) promotes the evaluation of process parameters and their interactions on response variables (Lu et al. 2013). It is worth combining with the application of SUMAE to extract phenolic compounds from walnut flour.

The objective of this study is to (a) optimize the phenol extraction process from WF by SUMAE with employing central composite design (CCD) and RSM; (b) investigate the effect of different parameters, including ultrasonic power, microwave power, extraction temperature, and extraction time; (c) identify the phenolic compounds in walnut flour extracts by LC–MS.

## Materials and methods

### Materials and chemicals

Walnut flour was donated by ZhaoTong Honglian Ltd., Yunnan, China, and stored in a freezer at −20 °C until the experiment, which was remained after cold pressed for walnut oil production with walnut kernels of a hybrid variety Yunxin of *Juglans regia* L. and *J. sigillata* Dode (Juglandaceae), identified by Professor Lu Bin in Yunnan Academy of Forestry. Folin–Ciocalteu reagent was purchased from Sigma (St. Louis, MO). Other chemicals (analytical grade) were from Beijing Chemical Co., Ltd. (Kunming, China).

### Simultaneous microwave/ultrasound-assisted extraction

The simultaneous microwave/ultrasound-assisted extraction (SMUAE) apparatus (DX100, MUAEA) was purchased from Nanjing Xian-ou Machine Co. Ltd. (Shanghai, China). The phenolic compounds extraction was performed using 60% methanol solution in the SMUAE apparatus. After the extraction, the extract was centrifuged at 10,000 rpm for 10 min and the supernatants were collected and then filtered with a 0.22 μm filter membrane. The samples were preserved at −20 °C prior to use.

### Detection of total phenol yield

The total phenol yield (TPY) of the WF extract was detected with a Folin–Ciocalteu assay (Shukla et al. 2012) with minor modifications (Wang et al. 2013). Briefly, 0.1 mL of sample was mixed with 1.9 mL of pure water and 1.0 mL of Folin–Ciocalteu reagent and reacted for 5 min. Then 1.0 mL of 20% Na_2_CO_3_ was added and reacted for 1 h at 25 °C. Gallic acid was used as the comparative standard. The absorbance was measured at 765 nm in an Ultrospec 2100 Pro UV–Vis spectrophotometer (Amersham Pharmacia Biotech Biochrom Ltd., Holliston, MA). The results were expressed as mg of gallic acid equivalents per 1 g walnut flour (mg GAE/g).

### Experimental design

To obtain the optimum extraction condition for WF, central composite design (CCD; Design Expert software, V 8.0.6, Stat-Ease Inc., Minneapolis, MN) was applied to the experimental design, data analysis and model building. Six replicates at the centre point (α = 1.5) were chosen as experimental points. The central points were used to check the reproducibility and stability of the results. The tests were conduct in randomized manner to guard against systematic bias. Based on the preliminary tests, a total of 30 runs from CCD were employed to optimize the main extraction conditions including the ultrasonic power (*X*_1_), microwave power (*X*_2_), extraction temperature (*X*_3_) and extraction time (*X*_4_) as shown in Table 1. The TPY (mg GAE/100 g) was the response variable *Y*.

**Table 1.** Central composite experimental design with the independent variables and comparison of observed data with predicted for the responses.

	Factors	TPY (mg GAE/g)				
No.	*X*_1_ (Ultrasonic, W)	*X*_2_ (Microwave, W)	*X_*3*_* (°C)	*X*_4_ (min)	Actual	Predict
1	40.00	150.00	30.00	2.00	19.01	18.99
2	120.00	150.00	30.00	2.00	21.37	21.15
3	40.00	450.00	30.00	2.00	20.90	20.85
4	120.00	450.00	30.00	2.00	23.23	23.30
5	40.00	150.00	50.00	2.00	25.12	24.82
6	120.00	150.00	50.00	2.00	28.73	28.51
7	40.00	450.00	50.00	2.00	27.26	27.44
8	120.00	450.00	50.00	2.00	31.33	31.42
9	40.00	150.00	30.00	8.00	23.41	23.27
10	120.00	150.00	30.00	8.00	27.75	27.47
11	40.00	450.00	30.00	8.00	21.80	21.92
12	120.00	450.00	30.00	8.00	26.15	26.40
13	40.00	150.00	50.00	8.00	23.57	23.40
14	120.00	150.00	50.00	8.00	29.11	29.12
15	40.00	450.00	50.00	8.00	22.63	22.81
16	120.00	450.00	50.00	8.00	28.90	28.82
17	20.00	300.00	40.00	5.00	25.80	25.87
18	140.00	300.00	40.00	5.00	31.80	32.00
19	80.00	75.00	40.00	5.00	27.33	28.17
20	80.00	525.00	40.00	5.00	29.90	29.34
21	80.00	300.00	25.00	5.00	28.08	28.20
22	80.00	300.00	55.00	5.00	34.23	34.38
23	80.00	300.00	40.00	0.50	26.01	26.27
24	80.00	300.00	40.00	9.50	27.53	27.54
25	80.00	300.00	40.00	5.00	34.90	34.10
26	80.00	300.00	40.00	5.00	34.43	34.10
27	80.00	300.00	40.00	5.00	33.80	34.10
28	80.00	300.00	40.00	5.00	34.57	34.10
29	80.00	300.00	40.00	5.00	34.50	34.10
30	80.00	300.00	40.00	5.00	32.90	34.10

### Identification of phenolic components by LC–MS

The phenolic components in the extracts obtained with the optimum condition were tentatively identified by LC–MS. The chromatographic separation was performed using an Agilent 1290 infinity series UHPLC instrument (Agilent Technologies, Santa Clara, CA) equipped with a quaternary pump (G4204A, USA), a de-gasser, a diode-array detector (G4212B, USA), an autosampler (G4226A, USA) and a column compartment (G1316C, USA). The chromatographic separation was achieved on an Agilent Zorbax SB C18 column (4.6 × 250 mm, 5 μm particle size) from Agilent Technologies. Acidified water (1% methanoic acid, v/v) and methanol were used, respectively, as mobile phases A and B.

The HPLC was programed to elute with 10% B for 5 min, a gradient elution of B from 10% to 90% from 5 to 50 min, and finally an isocratic elution of 90% B to 60 min. The flow rate was set at 0.5 mL/min throughout the elution. The column temperature and the injection volume were 30 °C and 10 μL, respectively. UV–Vis absorption was monitored by DAD at 280 nm. Mass spectrometric studies were carried out on a quadrupole time-of-flight (Q-TOF) high-resolution mass spectrometer (Q-TOF LC/MS 6540 series, Agilent Technologies, Santa Clara, CA) coupled with electrospray ionization (ESI). The detection was performed in positive ion mode with spectra acquired over a mass range from *m/z* 100 to 2000. The ESI–MS parameters were optimized as follows: capillary voltage was set at +4.5 kV, and nitrogen was used as the drying gas (180 °C, 4.0 L/min). The data were acquired using Agilent Mass Hunter Workstation software.

## Results and discussion

### Fitting the model

Response surface methodology was used to optimize the parameters of walnut flour extraction for TPY, including ultrasonic power (*X*_1_), microwave power (*X*_2_), extraction temperature (*X*_3_) and extraction time (*X*_4_), as shown in Table 1.

The statistical analysis of the regression model was evaluated by *F*-test. Table 2 summarizes the response surface polynomial model and the analysis of variance (ANOVA) performed to detect if the models fit. For any term in the model, a larger regression coefficient and a smaller *P*-value would indicate a more significant effect on the relative response variables (Teng et al. 2011).

**Table 2.** Analysis of variance **(**ANOVA) for the response surface polynomial model.

Source	SS	DF	MS	*F*-value	*P*-value
Model	625.46	14	44.68	155.52	<0.0001
Linear					
* X*_1_ (Ultrasonic, W)	85.52	1	85.52	297.71	<0.0001
* X*_2_ (Microwave, W)	3.11	1	3.11	10.82	0.0050
* X*_3_ (°C)	87.14	1	87.14	303.34	<0.0001
* X*_4_ (min)	3.64	1	3.64	12.68	0.0028
Interaction					
* X*_1_*X*_2_	0.084	1	0.084	0.29	0.5958
* X*_1_*X*_3_	2.34	1	2.34	8.13	0.0121
* X*_1_*X*_4_	4.12	1	4.12	14.35	0.0018
* X*_2_*X*_3_	0.58	1	0.58	2.03	0.1750
* X*_2_*X*_4_	10.32	1	10.32	35.91	<0.0001
* X*_3_*X*_4_	32.57	1	32.57	113.38	<0.0001
Lack of fit	1.69	10	0.17	0.32	0.9395
Pure error	9.17	4	2.29		
Total	2.62	5	0.52		
*R*^2^	0.9932				
*R*_adj_^2^	0.9868				

The Fisher’s *F*-test had a high model *F*-value (*F* = 625.46) and a low *P*-value (*P <* 0.0001), demonstrating that the models were highly significant. The resulting second-order polynomial model adequately represented the observed data with a *R*^2^ of 0.9932 for the TPY responses, which indicated a good correlation between the actual and predicted values. The adjusted coefficient (*R*^2^_Adj_) value was 0.9868 for TPY, which denoted that most of the variation could be predicted by the models. Additionally, the non-significant lack-of-fit test (*P* > 0.9) agreed with the goodness fit for the model. These results demonstrated that the response surface model could be used to predict the responses.

### Effect of extraction parameters on total phenol yield

The total phenol yield of the walnut flour phenol extract obtained by SMUAE based on CCD was shown in Table 1. Multiple regression analysis was performed on the experimental data, and the coefficients of the model were evaluated for significance. The factors of ultrasonic power (*X*_1_), microwave power (*X*_2_), extraction temperature (*X*_3_) and time (*X*_4_) in the TPY linear model were significant (*P <* 0.01) for the first-order main effect as well as in their interactive effects (*X*_1_*X*_3_*, X*_1_*X*_4_*, X*_2_*X*_4_, and *X*_3_*X*_4_). The polynomial equation of the relationship between the factors and the predicted response is given below:

$$\begin{array}{l} Y=34.10+2.04X_{1}+0.39X_{2}+2.06X_{3}+0.42X_{4}+0.073X_{1}X_{2}\\ +0.038X_{1}X_{3}+0.51X_{1}X_{4}+0.19X_{2}X_{3}-0.80X_{2}X_{4}-1.43X_{3}X_{4}\\ -2.30X_{1}^{2}-2.38X_{2}^{2}-1.25X_{3}^{2}-3.20X_{4}^{2} \end{array}$$

The linear coefficients for all factors (independent variables) were positive but negative for the quadratic coefficients, indicating that first-stage increases in the factors would be concomitant with increases in the response (dependent) variable TPY and vice versa; the second-stage increases in the factors would decrease TPY.

**Figure 1.**
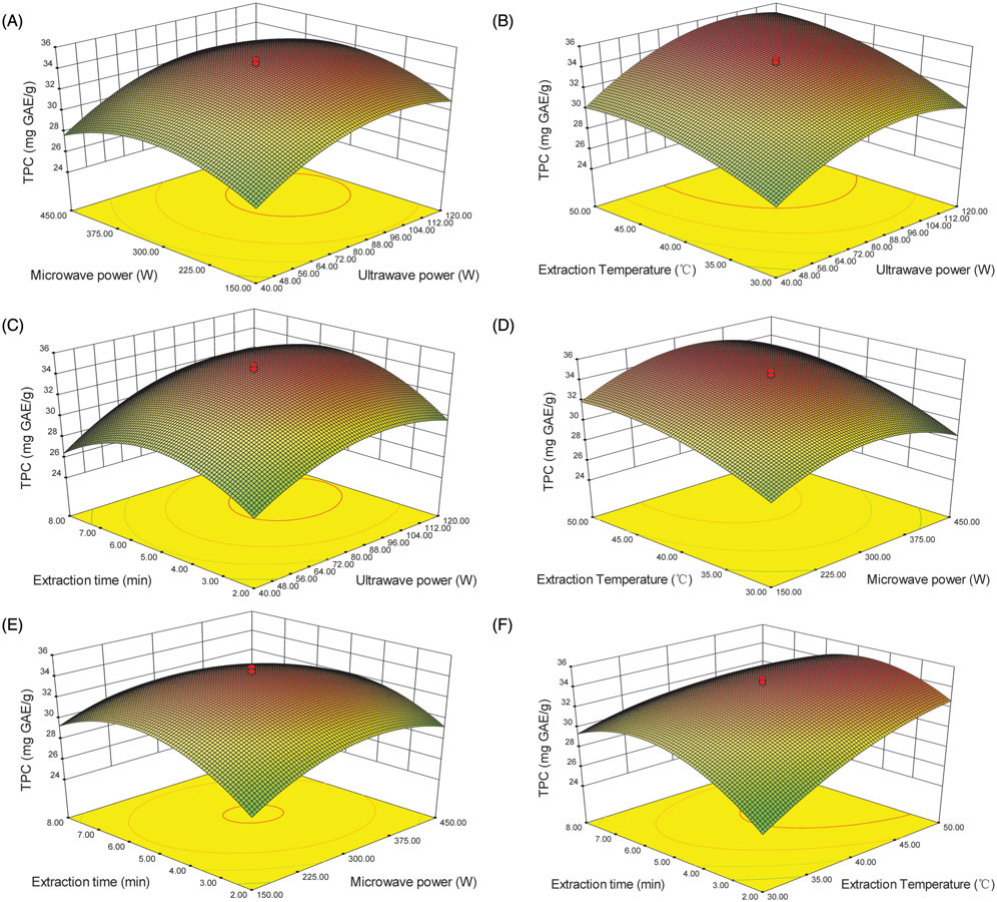
Response surface plots (3 D) showing the effect of different extraction parameters on the total phenolic yield (TPY). (A) Ultrasonic power and microwave power; (B) ultrasonic power and extraction temperature; (C) ultrasonic power and extraction time; (D) microwave power and extraction temperature; (E) microwave power and ethanol concentration; (F) extraction temperature and extraction time.

To observe the effects of simultaneous ultrasound/microwave-assisted extraction on TPY from walnut flour, 3 D surface plots (Figure 1(A–F)) were drawn on the basis of the equation, with each plot representing one pair of independent variables to investigate their interaction effect on TPY. Figure 1(A) shows the effects of microwave power and ultrasound power on the TPY. When the microwave power was fixed, the TPY increased with the increase in ultrasonic power until reaching the maximum and then decreased. Similarly, the increase in microwave power with the ultrasonic power fixed increased TPY to a peak, and then decreased it. Similar trends also existed for other interactions of factors (Figure 1(B–F)). From the above observations, with other factors fixed, TPY increased during the first stage with the increases in ultrasound power from 40 to 100 W, microwave power from 150 to 300 W, extraction temperature from 30 to 45 °C, extraction time from 2 to 5 min; inversely, TPY decreased in the second stage with excessive administration of ultrasound power from 100 to 120 W, microwave power from 300 to 450 W, extraction temperature from 45 to 55 °C, and extraction time from 5 to 8 min. The inversion of TPY under these excessive conditions, also noticed during our preliminary study, might be related to the degradation of bioactive substances due to the tear effect of dipolar rotation for microwave energy or/and ultrasound (Hayat et al. 2010; Pingret et al. 2013; Nour et al. 2016). In addition, the combined effects of oxidation during the extraction process and the interaction of phenolic and non-phenolic compounds such as sugar and fatty acid interactions (Pingret et al. 2013) might have lowered the TPY in the SUMAE extracts.

### Optimization and verification of the model

From solving the equation, the optimal conditions for polyphenol extraction from WF were obtained as follows: ultrasonic power of 93.5 W, microwave power of 294.38 W, extraction temperature of 43.38 °C and time of 4.33 min. Compared with the conventional extraction of phenolic compounds, SUMAE not only drastically reduced the extraction time at a mild temperature but also predicted a maximum TPY value of 34.91 mg GAE/g. The higher TPY when using SUMAE could be attributed to the microwave ability to penetrate the cell matrix and its interaction with polar molecules resulting in volumetric heating of the biomaterial, consequently leading to a pressure increase inside the plant cell. This increased pressure would lead to the breaking of cell walls and the release of phenolic analytes. In addition, the breakdown of larger phenolic compounds into smaller original molecules with their properties intact, as measured by Folin–Ciocalteu assay, could also provide higher TPY (Nayak et al. 2015).

To validate the model adequacy, experimental verification was performed using the optimum extraction conditions. The experimental mean value of TPY from WF was 35.12 ± 0.27 mg GAE/g, which was close to the predicted value of 34.91 mg GAE/g from the RSM model. The good consistency between the results indicated that the response model was adequate in reflecting the expected optimization and that the model could be used to optimize the process of phenolic compounds extraction from walnut flour. In addition, the TPY from WF was higher than those from apple pomace (40 °C, 40 min with TPY 5.55 mg GAE/g) (Pingret et al. 2012), potato peels (80 °C, 2 min with TPY 11.0 mg GAE/g) (Wu et al. 2012) and orange peels (40 °C, 30 min with TPY 2.758 mg GAE/g) (Khan et al. 2010). It is clear that walnut flour is a rich source of phenolic compounds that can be efficiently extracted by the optimized SUMAE method as an ingredient for functional foods and medicines.

### Identification of phenolic components by LC–MS

**Figure 2.**
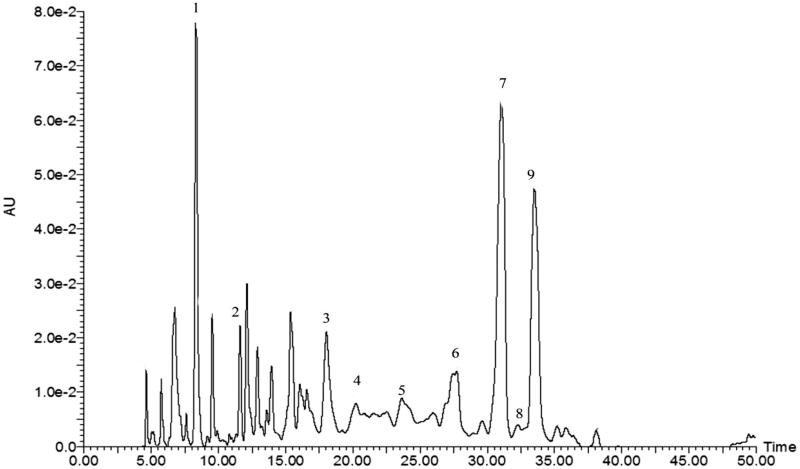
HPLC chromatogram of phenolic compounds in WF. Peak 1: Gallic acid; Peak 2: Vanillic acid glucoside; Peak 3: Digalloylglucose; Peak 4: Rutin; Peak 5: Cumaroylquinic acid; Peak 6: Glansreginin B; Peak 7: Glansreginin A; Peak 8: Chlorogenic acid; Peak 9: Ellagic acid.

The polyphenol substances were separated by HPLC, and the compositions for each peak were identified based on the mass to charge ratio (*m/z*) of the molecular ion and the characteristic fragment ions. Figure 2 shows the HPLC chromatogram of the phenolic compounds from walnut flour. The chromatogram peaks were identified by retention times and referencing the mass spectra to literature data as well as to certain authentic standards (rutin, gallic acid, ellagic acid, and chlorogenic acid) (Yang et al. 2013; Regueiro et al. 2014).

For phenolic compounds for which commercial standards were unavailable (vanillic acid glycoside, cumaroylquinic acid, and glansreginins A and B), the molecular ion and their differentiated MS/MS fragments were obtained by TOF analysis. The molecular ion 329.08 *m/z* gave 197.04 *m/z* as a fragment (for vanillic acid), and the difference between them is 162 *m/z* corresponding to a fragment of glucose. The molecular ion 483 *m/z* gave 169 *m/z* and 313 *m/z* as fragments of galloyl and galloylglucose; ion 337.09 *m/z* gave 163 *m/z* as a fragment (for coumaric acid); ions 592.20 *m/z* and 565.21 *m/z* both gave 403/343/241/197 as fragments, corresponding to the fragments previously described by Gomez-Caravaca et al. (2008) to be glansreginins A and B, respectively. Ultimately, nine phenolic components (gallic acid, vanillic acid glucoside, digalloylglucose, rutin, cumaroylquinic acid, glansreginin B, glansreginin A, chlorogenic acid, and ellagic acid) were tentatively identified, with their m/z ratios, MS/MS fragments, and compound names summarized in Table 3. The major phenolic components were glansreginin A, ellagic acid, and gallic acid with peak areas of 22.15%, 14.99% and 10.96%, respectively. The compound names in Table 3 are hyperlinked with PubChem to easily reference their functional data. Unidentified peaks presented a noticeable abundance of 33% total area; and are pending further study with better separation efficiency, greater sensibility of characteristic ions, and comprehensive polyphenol standard information.

**Table 3.** Phenolic compounds identified by LC-MS in WF extracts.

Peak	RT (min)	Area (%)	*m/z*	MS/MS fragments	Molecular formula	Proposed compound	Reference
1	8.40	10.96	170.12	125	C_7_H_6_O_5_	Gallic acid	Regueiro et al. (2014)
2	11.70	2.31	329.08	247/167	C_14_H_18_O_9_	Vanillic acid glucoside	Gomez-Caravaca et al. (2008)
3	18.04	5.16	483.08	313/169	C_20_H_20_O_14_	Digalloylglucose	Gomez-Caravaca et al. (2008)
4	20.01	2.65	609.14	301	C_27_H_30_O_16_	Rutin	Yang et al. (2013)
5	23.80	2.41	337.09	255/163	C_16_H_18_O_8_	Cumaroylquinic acid	Gomez-Caravaca et al. (2008)
6	27.82	5.32	565.21	403/343/241/197	C_24_H_38_O_15_	Glansreginin B	Gomez-Caravaca et al. (2008)
7	31.25	22.15	592.20	403/343/241/197	C_28_H_35_NO_13_	Glansreginin A	Gomez-Caravaca et al. (2008)
8	32.28	0.51	353.09	163	C_16_H_18_O_9_	Chlorogenic acid	Gomez-Caravaca et al. (2008)
9	33.61	14.99	300.99	–	C_14_H_6_O_8_	Ellagic acid	Gomez-Caravaca et al. (2008)

Our previous studies showed that walnut flour extracts inhibited pancreatic lipase activity *in vitro*; had a hypolipidemic effect on high-fat diet-induced obese mice (Shi et al. 2014); demonstrated a dose-dependent scavenging activity against hydrogen peroxide (H_2_O_2_), hydroxyl free radical (·OH), superoxide anion free radical (O^−2^·) and 2,2-diphenyl-1-picrylhydrazyl (DPPH) with IC_50_ values of 8.19, 151.43, 202.83, and 481.18 μg/mL, respectively (Liang et al. 2015); and improved glucose and lipid metabolism of postnatally monosodium glutamate (MSG)-induced obese mice, suppressing their weight gain and fat accumulation and increasing the activities of glutathione peroxidase and superoxide dismutase in their livers (Liang et al. 2017). In addition, some researchers have reported that ellagic acid has shown beneficial anti-atherogenic, anti-thrombotic, anti-inflammatory, and anti-angiogenic effects (Larrosa et al. 2010) in addition to antiviral activity (Chen et al. 2015). Gallic acid was responsible for anticancer effects (Faried et al. 2007) and exhibited anti-proliferative, pro-apoptotic and antitumorigenic effects against prostate carcinoma xenograft growth in nude mice (Kaur et al. 2009). The identified phenolic compounds in WF have been shown to have great benefits for health. Therefore, polyphenol-rich extracts from walnut flour can be used as a functional food ingredient and as a raw material for medicine.

## Conclusions

This paper optimized SUMAE extraction parameters based on an RSM model that could obtain the highest TPY from walnut flour. An ANOVA test indicated a significant univariate relationship for the independent variables so that the fitted model could be used for predicting responses (*R*^2^ = 0.9932, *P* < 0.0001). LC–MS results illustrated the relative peak areas of components in WF extracts in which nine phenolic compounds with several health benefits were identified. Thus, walnut flour, as a food oil by-product, can be used as a high-value functional food ingredient and a rich source of phenolic compounds for medicines.
